# Balance algorithm for cluster randomized trials

**DOI:** 10.1186/1471-2288-8-65

**Published:** 2008-10-09

**Authors:** Ben R Carter, Kerenza Hood

**Affiliations:** 1 South East Wales Trials Unit, Neuadd Meirionnydd, School of Medicine, Heath Park Campus, Cardiff University, CF14 4XN

## Abstract

**Background:**

Within cluster randomized trials no algorithms exist to generate a full enumeration of a block randomization, balancing for covariates across treatment arms. Furthermore, often for practical reasons multiple blocks are required to fully randomize a study, which may not have been well balanced within blocks.

**Results:**

We present a convenient and easy to use randomization tool to undertake allocation concealed block randomization. Our algorithm highlights allocations that minimize imbalance between treatment groups across multiple baseline covariates.

We demonstrate the algorithm using a cluster randomized trial in primary care (the PRE-EMPT Study) and show that the software incorporates a trade off between independent random allocations that were likely to be imbalanced, and predictable deterministic approaches that would minimise imbalance. We extend the methodology of single block randomization to allocate to multiple blocks conditioning on previous allocations.

**Conclusion:**

The algorithm is included as Additional file 1 and we advocate its use for robust randomization within cluster randomized trials.

## Background

An essential part of a randomized controlled trial (RCT) is the process of allocating units to treatment or intervention groups (arms). This is defined as randomization and is carried out to ensure that systematic bias is minimized during the selection stage [1-3] and that randomized rather than model based inference can be used for analysis. Randomization is carried out so that any differences found between the treatment arms can be argued as a genuine effect or due to chance. The key principles of randomization are to ensure each unit is allocated randomly and that future allocations are concealed. This ensures that knowledge of previous allocations does not allow prediction of those in the future. There are many ways to undertake randomization, with the most straightforward rolling an unbiased die with the treatment groups equally represented. However, in this simple example nothing prevents a heavy imbalance in terms of absolute number or baseline covariates to one of the treatment arms.

A solution to minimize the potential imbalance between treatment groups was originally reviewed by Box [4] then extended independently by Taves [5] and Pocock and Simon [6]. These authors detailed the steps implementing methods that dynamically randomized patients to treatment group, minimizing the imbalance between treatment baseline characteristics, rather than by chance. After minimization was introduced further authors introduced methods that were published but were felt to be suboptimal for the application [7,8]. Since then, the majority of views expressed have been in favour of widespread incorporation of minimization [9-11]. International guidance for the pharmaceutical industry has been the most notable critic of minimization and highlighted the lack of concealment [12], whereas other authors argue that in an RCT setting it is an additional administrative burden [13]. The Committee for Propriety Medicinal Products (CPMP) and at the International Conference on Harmonisation (ICH) for statistical principles in clinical trials it was advised that deterministic designs should be avoided and a random element included [12]. Many authors who have expressed reservation about minimization acknowledge that in settings where few units are needed to be randomized it can offer substantial benefits. Therefore minimization is an important consideration for cluster randomized trials where randomization occurs at the centre, rather than at the subject level. However, the key to minimization being accepted is the introduction of randomness to minimize the predictive power of those involved in a trial. This can be by masking and concealment to reduce the risk of allocations being known prior to randomization [14].

Cluster randomized trials may recruit all units prior to randomization which would allow baseline characteristics to be used to calculate the imbalance between treatment arms for each allocation allowing minimization with complete knowledge of baseline characteristics across the sample. Raab and Butcher introduced two criteria to evaluate randomization methods in cluster randomized trials across baseline covariates and in doing so described a simple imbalance measure between treatment arms [15]. Using this measure they generated allocations through randomization block designs which have been criticized for advance sequential randomization [16-18]. However, by delaying the allocation until all units within a block have been enrolled adequately deals with the issue of concealment [2,19,20]. Since the initial work of Raab and Butcher was reported other studies have used the methodology [21-23]. In a primary care study randomised by practice, this would mean all practices being identified and enrolled prior to randomization.

A natural extension of this is to consider blocks of units as they are enrolled. For a study in primary care this may mean practices which rapidly take up the offer of taking part in a study (and for whom the local ethical and governance arrangements are completed) for a first block, then those who take longer forming a second. With blocks structured pragmatically, it is important to balance between blocks as well as within.

At present no public domain software has been made available to calculate a within and between block imbalance measure using baseline covariate information. Our software is freely available through the R Software [24].

## Implementation

### Randomization of a single block of units

Where all units are fully identified in advance, a single block can be used for the study. The algorithm carries out a complete enumeration of all allocations in a two-treatment arm study. When the number of units within a block to be allocated is even, an equal number of units would be allocated into each of the treatment arms. For cases with an odd number of units within block a near equal allocation is generated between the two treatment arms. Once the set of possible enumerations has been generated the imbalance statistic is calculated using the baseline covariates for each allocation across the two treatment arms.

It should be remembered that allocating units within a two arm study design incurs a natural symmetry. Since the algorithm does not assign treatment arm, only 0 or 1 as a treatment arm code, these can be interpreted as either treatment arm. Thus, a design with the first half of units allocated to treatment arm 0 and subsequent allocated to 1 would be identical to the first half of units allocated to treatment arm 1 and latter to 0. Therefore, for single block designs the software always allocates the first unit into group 1.

The imbalance measure as calculated by Raab and Butcher [15] was coded -1 and 1 for the two treatment group, here it is equivalently coded within block using 0 and 1 calculated using: *x*_*ij *_which is a matrix of 0, and 1's denoting allocation to treatment arm for each unit (*i*) and allocation (*k*); and the matrix of equally weighted z-scores for the baseline factors noted as *w*_*ij*_; and can be written as:

(1) $$\begin{array}{cc} Balance=\sum_{j=1}^{\text{M}}{\left(\sum_{i=1}^{n_{1}}\left(x_{ik}w_{ij}\right)\right)}^{2} & \\ & \begin{array}{l} \begin{array}{cc} i=1,2,... & n_{1}, \end{array}\\ \begin{array}{cc} j=1,2,... & \text{M}, \end{array}\\ \begin{array}{cc} k=1,2,... & 2^{n_{1}}, \end{array} \end{array} \end{array}$$

where *x*_*ik *_is the *i*^th ^unit of the *k*^th ^allocation, *w*_*ij *_is the *i*^th ^unit of the *j*^th ^baseline covariate, n_1 _is the number of units allocated to the first block and M is the number of baseline factors.

The algorithm will provide a set of optimal allocations depending on the number of units to be randomized. In accordance with the principles of the ICH guidance on randomness it is recommended that the final design is sampled from a set of optimal allocations [12,14]. We have offered guidance to the minimum size of the sets required in Table 1. We use the 25% most optimal allocations for blocks with between 8 to 11 units or the optimal 100 allocations for blocks between 12 and 17 units and for larger block sizes with greater than 17 units we use the top 1,000 allocations. The size of the block affects the level of predictability and concealment, with smaller block sizes more susceptible to bias [25].

**Table 1:** The number of units, total allocation permutations and size of random component that the final design is selected from, partitioned into first and additional blocks

Units	Allocations	Random element for first block		Random element for additional blocks	
		Number	Percent	Number	Percent
6	20	-	-	7	35%
7	35	-	-	10	29%
8	70	10	29%	18	26%
9	126	18	29%	32	25%
10	252	32	25%	63	25%
11	462	58	25%	100	22%
12	924	100	22%	100	11%
13	1,716	100	12%	100	6%
14	3,432	100	6%	100	3%
15	6,435	100	3%	100	2%
16	12,870	100	2%	100	1%
				
17	24,310	100	1%	1,000	4%
				
18	48,620	1,000	4%	1,000	2%
19	92,378	1,000	2%	1,000	1%
20	184,756	1,000	1%	1,000	1%
21	352,716	1,000	1%	1,000	0%
22	705,432	1,000	0%	1,000	0%
23	1,352,078	1,000	0%	1,000	0%
24	2,704,156	1,000	0%	1,000	0%
25	5,200,300	1,000	0%	1,000	0%
26	10,400,600	1,000	0%	1,000	0%
27	20,058,300	1,000	0%	1,000	0%
28	40,116,600	1,000	0%	1,000	0%
29	77,558,760	1,000	0%	1,000	0%
30	155,117,520	1,000	0%	1,000	0%

Once the final allocated has been selected the choice of which group becomes intervention or control should be allocated randomly.

### Randomization of multiple blocks of units

As described above there are often practical reasons for using a number of blocks. Therefore, there is a desire to randomize smaller blocks as they become availbale. Computationally the enumeration of 20 practices balanced into a two arm design would lead to 184,756 possible allocations. Beyond 20 units the total number of enumerated allocations quickly becomes a computationally intensive problem, where the maximum number of units able to be randomized is dependent upon the amount of available RAM (Table 2). Therefore, it may be prudent to randomize in blocks to overcome these two difficulties. Smaller block sizes have a increased chance of selection bias through inadequate concealment, or inquisitive investigators [26], however if all units are enrolled prior to randomisation and informed at the same time of their allocation this is unlikely to be an issue.

**Table 2:** The maximum number of units able to be allocated, dependent on RAM specification and block number.

RAM	First block	Additional blocks
256 Mb	22	20
512 Mb	22	20
1024 Mb	24	22
2048 Mb	24	22

Second and subsequent blocks should be allocated using the selected design of earlier blocks. The structure of the input allocation from earlier blocks includes the same header with a single row of 0 and 1's allocating units into the two treatment arms.

For an even block size the allocation will be equally split between the two treatment arms (regardless of previous blocks). For odd block sizes the previous blocks allocations will be considered, since if previous blocks had equal number of units within each treatment arm, then a random number generator would allocate the greater number units to one of the arms. However, if the two arms had already been allocated a different number of units then the greater number of units would be automatically allocated to the lesser recruited treatment arm. For example, if block one allocated 13 units with 6 in arm 0 and 7 in arm 1, and block two were to allocate 15 units then 8 would be allocated to arm 0 and 7 to arm 1.

For each additional block the balance measure is conditional on the selected allocation of the first block, where *w*_*ij *_is the within block z-scores and the balance measure becomes:

(2) $$\begin{array}{cc} Additional\;Block\;Balance=\sum_{j=1}^{\text{M}}z_{jk}^{2}, & \text{since }z_{jk}=\sum_{i=n_{1}+1}^{n_{2}}\left(x_{ik}w_{ij}\right)|\sum_{i=1}^{n_{1}}\left(x_{ik}w_{ij}\right), \end{array}$$

where: n_2 _is the number of units allocated to the additional block.

The output provided from multiple block designs is similar a single block design. The differences in methodology between the allocation of the first and additional blocks are the following:

• if the block size is odd, the allocation of the larger number of units will depend on previous block allocations,

• the symmetry which existed in the first block, no longer exists in additional blocks, since the treatment arm code has already been allocated. This changes the number of units able to be allocated within a block (Table 1).

### The baseline covariate data

Numerical covariate information can be used directly in 'covariate_csv'. However, categorical data should be coded as below:

• If the factor has a natural ordering and would be deemed ordinal categorical then ordered scores should be considered for example: None, mild, moderate and severe disease levels could be coded within the data as 0,1,2,3. However, depending on the extent of the difference between the ordinal categories these might be coded alternatively on the log_2 _scale i.e. 0,1,2,4. We advise you to seek statistical and clinical advice for guidance before proceeding with these [26].

• If the factor lacks a natural ordering and is considered nominal categorical then the number of levels within the factor will need to be considered. Nominal categorical factors can be coded using orthogonal dummy variables which identify individual factor levels (Table 3).

**Table 3:** Orthogonal coding of dummy variables for nominal categorical factor levels using a single variable for 2 levels, two variables for 3 to 4 levels and three variables for 5 to 8 levels

Number of levels	Level	Var_1_	Var_2_	Var_3_
2	1	-1	N/A	N/A
	2	1		

3	1	-1	-1	N/A
	2	1	-1	
	3	-1	1	

4	1	-1	-1	N/A
	2	1	-1	
	3	-1	1	
	4	1	1	

5	1	-1	-1	-1
	2	1	-1	-1
	3	-1	1	-1
	4	-1	-1	1
	5	1	1	1

6	1	1	-1	-1
	2	-1	1	-1
	3	-1	-1	1
	4	-1	1	1
	5	1	-1	1
	6	1	1	-1

7	1	-1	-1	-1
	2	1	-1	-1
	3	-1	1	-1
	4	-1	-1	1
	5	-1	1	1
	6	1	-1	1
	7	1	1	-1

8	1	-1	-1	-1
	2	-1	-1	1
	3	-1	1	-1
	4	-1	1	1
	5	1	-1	-1
	6	1	1	-1
	7	1	-1	1
	8	1	1	1

• It should be remembered that units should be equally allocated amongst each of the levels of each factor. Therefore, it would be recommended to include factors with few levels and few factors with greater than two levels, this can often be achieved by aggregating related levels. However to code a factor with three of four levels can be carried out by implementing two variables in the covariates data sheet, or with 5 to 8 levels with 3 variables (see Table 3). To code a nominal factor called 'type of health professional' with three factors levels 'GP', 'Nurse' and 'Other' could be coded as the following: 'GP' (var_1 _= -1, var_2 _= -1), 'Nurse' (var_1 _= 1, var_2 _= -1) and 'Other' (var_1 _= -1, var_2 _= 1).

## Results

The software has been used within the PRE-EMPT study [27]. This is a study to evaluate the impact of training primary care health professionals in behaviour change counselling. The study was randomized at the practice level and recruited patients prior to their appointment with a GP or nurse and followed up 3 months later. Practices within block were all enrolled into the study, then randomized and then informed of their allocation. Two blocks of 14 and 15 were used respectively, to allow for differing rates of response from practices and approvals being gained.

Here we present data of 29 general practice surgeries where the first 14 (rows) were allocated in block 1, and the remaining 15 allocated to block 2 using 'covariate.csv' as the baseline covariates data. The baseline data included two covariates, the first general practice list size (the number of patients registered at that practice) and the Townsend deprivation index aggregated to the general practice level [28-30]. The allocations for block two was conditional on block one has been included as 'block_one_allocation.csv'.

**Figure 1:**
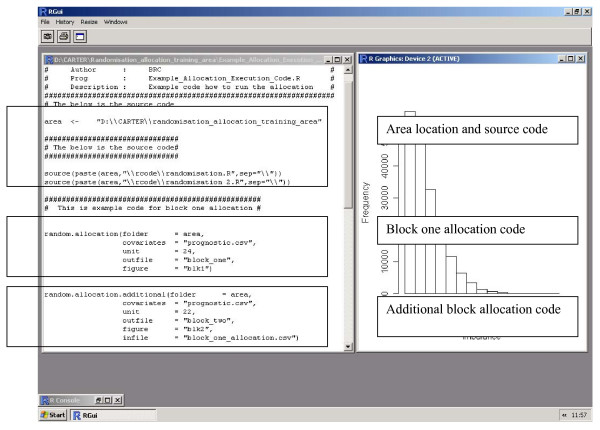
**A screen dump from 'Example_Allocation_Execution_Code.R'. **To allocate, change the area and submit the R-script.

To implement the randomization algorithm carry out the following:

1, check the system requirements in the availability and requirements section, ensuring that you have installed R version 2.4, or later,

2, create a main folder and a subsequent subfolder within this called 'rcode',

3, save "Example_Allocation_Execution_Code.R" and "covariate.csv" within the main folder, then "randomisation.R" and "randomisation 2.R" within the subfolder,

4, open R and update the location of the 'area', highlight and submit the area and source code (Figure 1)

5, to run the algorithm on block one, update the data within the block one allocation code and submit. Inversely to run later block allocations, update the data within the later block allocation code and submit this. Where the following variables are required (Figure 1):

folder, the location of the data, typically the same as the 'area';

covariates, the file containing the baseline covariate information;

unit, the number of units within the block;

outfile, the file produced containing the optimal set of enumerated allocations ordered by the imbalance statistic;

figure, the figure of the distribution of imbalance statistics across all enumeration allocations;

infile, the previously allocated block data (only required for blocks two or later).

**Figure 2:**
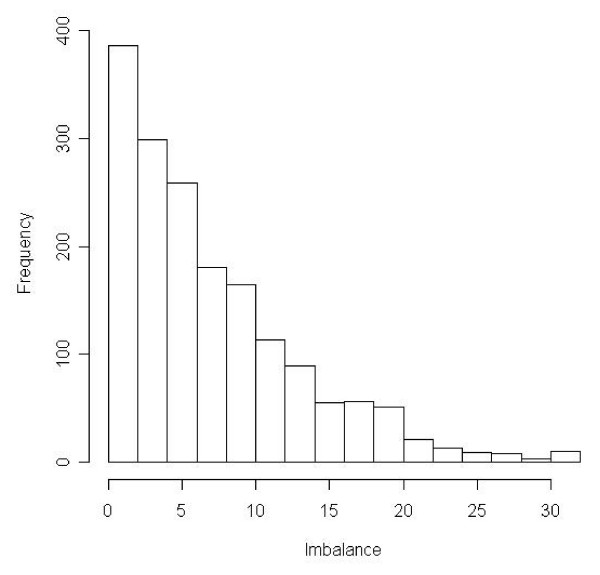
The distribution of imbalance statistics across each of the generated allocations for block one.

This will generate a file with the set of optimally balanced allocations ordered by the imbalance statistic called 'block_one.csv', where the rows are the allocations and the columns are the general practices as taken from the covariate file. A histogram that visualises the distribution of the imbalance statistics calculated from all allocations is shown in Figure 2.

From 'block_one.csv' one allocation was selected at random and treatment arm codes 0 and 1 were allocated to control and intervention arms (highlighted in 'block_one.csv'). Later the second block final allocation was selected and can be found as 'block_two.csv' (highlighted again). Final allocations for both blocks were random selectly by the independent statistician on the Trial Steering Committee.

Table 4 presents summary statistics for the two baseline covariates including the number randomized into each arm (n), the mean and standard deviation (sd) within block and across blocks. This shows that the two arms exhibit minimal evidence to suggest imbalance either within or between blocks.

**Table 4:** Summarised baseline information from the randomised general practice surgeries for the two covariates partitioned into treatment group

		List-Size		Townsend Deprivation Index	
	(n)	mean	sd	Mean	sd
Block 1					
Control	7	1,483	970	0.9	2.87
Intervention	7	1,504	570	1.1	1.70

Block 2					
Control	7	2,080	929	1.5	3.58
Intervention	8	1,843	1112	0.7	5.26

Block 1 & 2					
Control	14	1,781	964	1.2	3.13
Intervention	15	1,685	888	0.9	3.89

## Discussion

The allocation of the first block involves the full enumeration of each design. These are used to calculate an imbalance measure between treatment arm [Equation 1]. Each additional block was randomized conditional on the previously allocated design as a fixed starting point [Equation 2]. In doing so the algorithm not only provides access to software but extends the work of Raab and Butcher [15] by allowing multiple blocks to be randomized and ensures that allocations are balanced between the two treatment arms.

A minimum number of recruited units available for randomization is crucial to maintain concealment and allocate a minimally imbalanced design. We propose that given the symmetry and importance of the first block this should be randomized with at least eight units. However, this could be reduced for subsequent blocks to six units, see Table 1 for more details of the total number of allocations that would be included in the allocation sets.

We advocate the allocation is selected at random from the set of allocations with the smallest imbalance statistic. Typically when using larger block sizes a random sample should be made from the 1,000 optimally balanced designed. However, for smaller block sizes fewer allocations should be used to randomly select from (Table 1). The set sizes were determined pragmatically and dependent on block size, but we recommend that further work is carried out to quantify their effect, as we recognise these will effect the degree of randomness introduced.

It would be envisaged that these algorithms are not only used by statisticians, but allow medical researchers with minimal access to a statistician an invaluable tool to help randomize their trials in a robust way. In the primary care setting these algorithms have already been used to randomize centres within cluster randomized trials. In future the algorithms could be extended to incorporate improved efficiency to deal with larger block sizes. Furthermore, we will weight the balance measure by practice recruitment for those recruited practices to deal with under recruiting practices and examine the impact on future block allocations.

For details of a wide range of alternative randomization software algorithms see Professor Martin Bland's randomization software services pages [31].

## Conclusion

The software is important for multi disciplinary teams needing to address the issues surrounding randomization. It allows the inclusion of information from baseline covariates to influence the allocation of the units to treatment groups, without disclosing or causing untoward doubt to the concealment.

It is intended these algorithms are an easy to use and convenient tool to be used by researchers who wish to minimize imbalance between treatment arms across multiple baseline stratification variables ensuring that ICH guidance is adhered to.

## Competing interests

The authors declare that they have no competing interests.

## Authors' contributions

BC undertook the literature review, extended the methodology, wrote the R-scripts and drafted the manuscript. KH carried out the original randomization work within the trials unit and reviewed the manuscript. Both authors have seen and approved the final manuscript.

## Availability and requirements

Project name: : Cluster randomization allocation algorithm,

Operating system(s) : Windows 95, Windows 2000/ME or Windows XP (Vista untested),

Programming language : R,

License : Scripts provided free for non-commercial use, with absolutely no warranty,

Other requirements : R 2.4.0 (Released 3^rd ^of October, 2006),

: R 2.6.1 is free to download and install [24],

: Minimum 256 Mb RAM (Table 2),

: 100 Mb Hard-disk space,

Restrictions : Commercial organisations should contact the author prior to use.

## Pre-publication history

The pre-publication history for this paper can be accessed here:



## Supplementary Material

Additional File 1 **Cluster randomization allocation algorithm version 1.** Algorithms scripted in R to provide robust cluster randomization. Click here for file
